# Addressing Research Gaps in Early Childhood Caries: A Comprehensive Review

**DOI:** 10.3390/dj14040196

**Published:** 2026-03-30

**Authors:** Anthony Yihong Cheng, Faith Miaomiao Zheng, Jieyi Chen, Chun Hung Chu

**Affiliations:** 1 Faculty of Dentistry, The University of Hong Kong, Hong Kong SAR 999077, China; ayhcheng@connect.hku.hk (A.Y.C.); faithzh@hku.hk (F.M.Z.); 2 Hospital of Stomatology, Guanghua School of Stomatology, Sun Yat-Sen University, Guangzhou 510055, China

**Keywords:** caries, oral health, prevention, children, childhood, preschool

## Abstract

**Background**: Early childhood caries (ECC) is one of the most common chronic diseases in children and remains unevenly distributed across populations. It is associated with pain, impaired function, and long-term health consequences. Although advances have been made in understanding its aetiology and prevention, important gaps in evidence limit progress in prevention, early detection, and equitable care. **Objective**: To examine current evidence on ECC and identify key research gaps across biological, behavioral, social, and health system domains. **Methods**: This narrative review draws on peer-reviewed literature addressing ECC epidemiology, pathogenesis, risk factors, diagnosis, management, and service delivery. The literature was examined to identify areas where evidence is limited, inconsistent, or insufficient to inform clinical practice and public health policy. **Results**: Research on ECC remains uneven across levels. Longitudinal evidence linking microbiome dynamics, host susceptibility, and lesion progression is limited, restricting causal understanding. Genetic and epigenetic contributions are incompletely defined, particularly in diverse populations. Although socioeconomic gradients are well established, integrative models connecting structural determinants with biological mechanisms are scarce. Emerging diagnostic tools, including biomarkers and artificial intelligence, lack robust evidence demonstrating improved clinical or behavioral outcomes. Implementation research addressing scalability, cost-effectiveness, and equity impact is underdeveloped, especially in low-resource settings. Long-term systemic and developmental consequences of ECC remain insufficiently characterized. **Conclusions**: Addressing ECC requires integrated and equity-oriented research frameworks that bridge biological, social, diagnostic, and implementation domains. Clarifying these gaps is essential to inform coherent prevention strategies and reduce persistent disparities in child oral health.

## 1. Introduction

### 1.1. Background and Scope

Early childhood caries (ECC) is defined as the presence of one or more decayed, missing, or filled primary teeth in children under six years of age [1]. It affects an estimated 530 million children worldwide [2], making it one of the most prevalent chronic conditions of childhood. ECC is associated with pain, infection, impaired nutrition, disrupted sleep, and reduced quality of life, and it may influence school participation and general health [3]. Children affected by ECC are at increased risk of caries in permanent dentition [4].

Importantly, ECC occurs during a critical period of growth and development, when biological vulnerability and social dependency intersect. ECC is widely recognized as preventable. Its pathogenesis reflects interactions among cariogenic biofilms, frequent exposure to fermentable carbohydrates, fluoride availability, host susceptibility, and oral health behaviors [5,6]. These processes operate within broader social and structural contexts, including caregiver education, food environments, health literacy, and access to preventive services [7]. Socioeconomic gradients in ECC prevalence are consistently reported across countries, although their magnitude varies by setting [8]. As a result, ECC often represents one of the earliest manifestations of health inequality in childhood.

Despite decades of research on risk factors and prevention, the burden of ECC remains high. In many regions, prevalence has not fallen in line with advances in knowledge or clinical guidelines, suggesting that evidence is not being effectively translated into practice. ECC arises from multiple interacting factors. Biological mechanisms, such as microbiome shifts and enamel vulnerability, combine with feeding practices, sugar exposure, and oral hygiene. These are embedded in wider social and structural conditions, including poverty, stress, access to care, and health-system design. Yet these domains are often studied separately, so we lack insight into how they interact over time to shape onset, progression, and recurrence.

Key gaps remain. Evidence linking the microbiome, diet, and family or community context is limited. Data from low-resource settings are uneven, restricting context-appropriate strategies [9]. Novel tools for early detection and risk assessment show promise but require validation in diverse populations and clear pathways for routine use. Without addressing these gaps, ECC prevention will continue to target isolated risks rather than the systems that sustain disease.

The aim of this review is to synthesize current evidence across biological, behavioral, diagnostic, and health-systems domains to identify priority research gaps and outline directions for more coherent, integrated, and effective approaches to ECC management.

### 1.2. Methods of Article Selection

This narrative review was informed by focused searches of PubMed, Scopus, and Web of Science for English-language publications between January 2015 and September 2025. Searches centered on “early childhood caries” and related terms (e.g., “severe early childhood caries,” “preschool children,” “primary dentition”), combined with keywords reflecting the thematic domains addressed in this review, including microbial ecology, dietary and sugar exposure, fluoride and preventive strategies, diagnostic methods, genetic and epigenetic factors, social and structural determinants, inequalities, and implementation approaches. Seminal studies published prior to 2015 were consulted where necessary to provide conceptual background.

Peer-reviewed original studies, systematic reviews, and meta-analyses were prioritized. Publications focused exclusively on permanent dentition, case reports, commentaries, and articles outside the thematic scope of early childhood caries were not considered. Articles were selected based on their relevance to the conceptual framework of this review and their contribution to identifying research gaps within each domain.

## 2. Multilevel Determinants and Mechanisms

### 2.1. Microbiome and Ecological Dynamics

**Figure 1:**
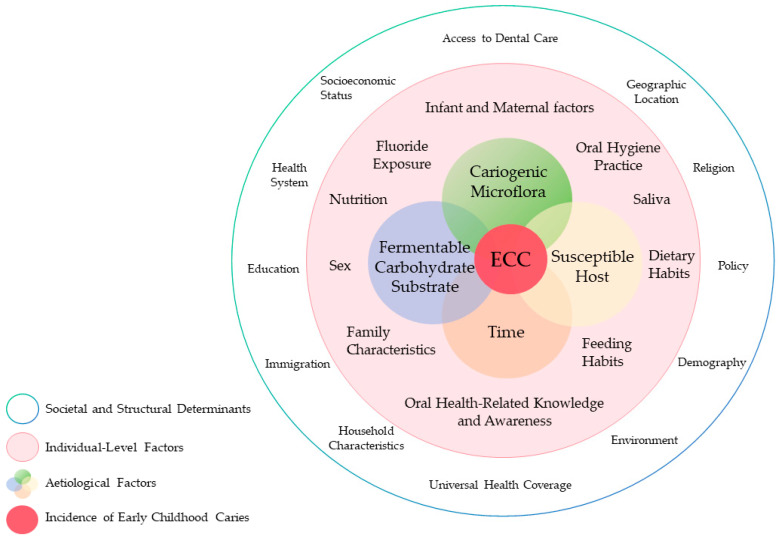
The multifactorial aetiology and risk factors of early childhood caries.

Figure 1 illustrates the multifactorial framework of ECC. The condition is understood as a biofilm-mediated process involving interactions among microorganisms, fermentable carbohydrate exposure, host susceptibility, and time [5,6]. The oral microbiome functions as a mediator of ecological imbalance [9]. Although *Streptococcus mutans* and other acidogenic bacteria have long been associated with lesion development [10], and fungal species such as Candida albicans have been linked to severe forms of ECC [11], these associations alone do not fully explain disease initiation or progression.

A major limitation of current evidence is its reliance on cross-sectional designs [9]. These studies describe microbial composition at a single time point and cannot determine whether microbial shifts precede lesion development or follow ecological change. Data linking microbial succession to lesion activity are limited. As a result, microbial markers of risk remain poorly defined.

Dietary exposure represents a major ecological pressure shaping biofilm composition. Frequent intake of free sugars lowers plaque pH and selects for acidogenic and aciduric communities [12,13,14]. The frequency and timing of sugar consumption, including repeated snacking and nocturnal feeding, are consistently associated with ECC risk [15,16]. However, many microbiome studies do not integrate detailed dietary assessment, salivary parameters, and clinical activity measures within the same analytical framework. As a result, the interaction between ecological pressure, microbial adaptation, and lesion activity over time remains incompletely characterized.

Beyond bacterial communities, interkingdom interactions warrant further investigation. Experimental and clinical studies suggest that fungal–bacterial cooperation may enhance biofilm virulence [17], yet the magnitude and clinical relevance of these interactions in diverse populations remain unclear. The contribution of oral viruses to ecological stability or dysbiosis in ECC has been minimally explored [18]. Host factors, including salivary flow and buffering capacity, may modify microbial selection pressures [19], intervention studies targeting microbial modulation are small and heterogeneous [20,21].

**Figure 2:**
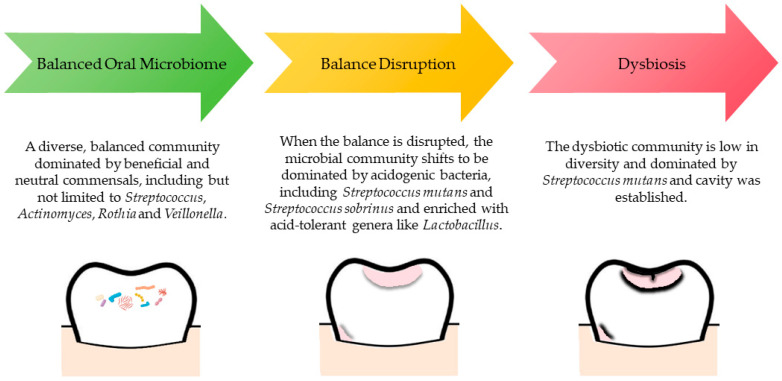
The microbial shift in the development of early childhood caries. ECC development reflects a shift from a diverse, balanced oral microbiome to a low-diversity, acidogenic and aciduric communities that drives demineralization and cavitation.

Figure 2 shows the microbial shift in ECC development [22]. Advances in metagenomic and metabolomic approaches provide tools to examine microbial function and ecological adaptation with greater resolution [23,24]. However, evidence is insufficient to determine whether altering microbiome composition changes long-term disease trajectories beyond established fluoride-based prevention. Overall, a key research gap lies in the absence of integrative models that combine microbial, dietary, host, and clinical data to establish causal pathways.

### 2.2. Genetic and Epigenetic Susceptibility

Genetic factors contribute to ECC, but their interplay with environmental influences remains poorly understood. Variations in genes linked to enamel formation may weaken tooth structure, increasing cavity risk [25]. However, not all children with these genetic variants develop ECC, suggesting that diet, oral hygiene, or other external factors modify genetic susceptibility. Despite this complexity, most studies focus on isolated genetic markers rather than how genes interact with behaviors like feeding practices [25,26].

A major research gap involves epigenetic mechanisms, which regulate gene expression without altering DNA. Maternal nutrition or stress during pregnancy could “program” a child’s oral microbiome or immune response through epigenetic changes [27]. Early-life antibiotic use might disrupt oral bacteria balance, increasing caries risk through epigenetic pathways [28]. These connections are rarely explored, leaving a critical gap in understanding how early exposures shape lifelong vulnerability. Another challenge is the lack of biomarkers for personalized prevention. While saliva tests can measure cavity-causing bacteria [29], they do not account for genetic or epigenetic profiles. Identifying biomarkers that predict high-risk children could enable tailored interventions, such as targeted fluoride treatments or dietary counseling. Current prevention strategies often take a one-size-fits-all approach, missing opportunities to protect genetically susceptible children before decay begins.

Future research should prioritize genome-wide association studies in diverse populations. Most genetic studies on ECC involve high-income cohorts, limiting insights into global susceptibility patterns [30]. Including underrepresented groups could reveal protective genetic variants or unique environmental interactions in different regions. Studies tracking epigenetic changes from pregnancy through childhood are equally vital [31]. For example, following mothers with gestational diabetes could clarify how maternal metabolic health influences a child’s oral epigenome and caries progression.

Interdisciplinary collaboration will accelerate progress. Geneticists, dentists, and public health experts must work together to translate findings into practical tools. Combining genetic risk scores with environmental data could improve early identification of high-risk children [30]. Meanwhile, epigenetic insights might inform prenatal counseling, encouraging behaviors that mitigate inherited risks. By unraveling the genetic and epigenetic roots of ECC, researchers can move closer to precision prevention—a critical step toward reducing disparities in this widespread childhood disease.

### 2.3. Social and Structural Determinants

Higher prevalence is reported among children from low-income households, families with lower caregiver education, and marginalized communities, although the magnitude varies by context [7,32]. Socioeconomic conditions influence diet quality, fluoride exposure, oral hygiene routines, and access to care [33]. Limited health literacy may delay care-seeking and affect perceptions of primary teeth [34].

While these associations are established, the pathways linking structural disadvantage to biological disease processes are not well specified. Factors such as food insecurity, housing instability, and neighborhood resource distribution are hypothesized contributors but are rarely incorporated into ECC research. Consequently, the mechanisms through which structural inequities translate into sustained ecological imbalance remain unclear.

Cultural beliefs and caregiving norms may shape dietary exposure, hygiene routines, and perceptions of primary teeth, thereby influencing biological risk pathways [35,36]. For example, practices such as sharing utensils with infants or providing sweetened comfort foods may increase sugar exposure, while multigenerational caregiving structures may offer protective potential [35,36]. However, empirical models that quantify how culturally embedded practices translate into ecological imbalance or lesion progression remain limited, and the mechanisms linking cultural context to measurable disease processes are insufficiently specified.

Policy environments may also influence exposure patterns and service access. Evidence on how fiscal policies, fluoridation regulations, or insurance coverage affect ECC distribution across socioeconomic groups remains limited [37,38]. Comparative analyses across regions are scarce, and policy impacts are rarely evaluated using equity-focused designs.

Overall, while socioeconomic gradients in ECC are established, integration of structural, cultural, and biological data within unified analytical models remains limited. Addressing this gap is necessary to clarify how social context translates into measurable disease processes.

## 3. Diagnostic Approaches

### 3.1. Conventional Examination and Adjunctive Methods

Visual–tactile examination remains the primary method for diagnosing ECC in clinical practice. It is reliable for detecting cavitated dentin lesions but less consistent for early, non-cavitated enamel lesions [39]. Early lesions may appear as subtle white-spot changes and are influenced by lighting, drying, time constraints, and child cooperation in preschool settings [40]. Examiner judgement also introduces variability, particularly where calibration is limited.

Adjunctive tools, including intraoral radiography and fluorescence-based devices, may support lesion detection [41]. Radiography improves identification of dentin involvement but offers limited additional value for early enamel-stage changes. Few studies examine whether adjunctive methods alter treatment decisions or improve outcomes in primary dentition.

A key gap concerns clinical relevance. Evidence linking improved early detection to changes in lesion progression, treatment intensity, or avoidance of overtreatment remains limited. Comparative studies conducted under routine practice conditions are scarce.

Beyond technical performance, the broader consequences of early diagnosis in primary dentition remain insufficiently examined. Evidence is limited regarding whether early identification of non-cavitated lesions improves caregiver engagement in preventive behaviors, enhances adherence to fluoride use, or modifies treatment-seeking patterns. Conversely, the potential for diagnostic labeling to increase parental anxiety or contribute to unnecessary intervention has not been well studied. The behavioral and clinical implications of early detection therefore remain uncertain.

### 3.2. Biomarkers and Digital Detection Technologies

Salivary biomarkers have been investigated as non-invasive indicators of early ECC. Microbial profiles, host proteins, and inflammatory markers may signal ecological imbalance before cavitation becomes clinically obvious [29,42]. They therefore hold promise for very early risk stratification. However, most studies are cross-sectional, exploratory, and use small, convenience samples. Standardized collection and analysis protocols are lacking, and reproducibility across age groups, dentitions, and populations has not been established. Longitudinal evidence linking specific salivary profiles to lesion initiation and progression is particularly limited.

Artificial intelligence-based image analysis systems have been developed to assist detection [43,44]. Current evidence is heterogeneous and often derived from retrospective or controlled datasets [45]. External validation in routine preschool environments, including low-resource settings, is sparse. Few studies assess whether AI-assisted detection improves diagnostic agreement, appropriateness of management decisions, or patient-centered outcomes compared with conventional visual–tactile examination and radiograph [46].

**Figure 3:**
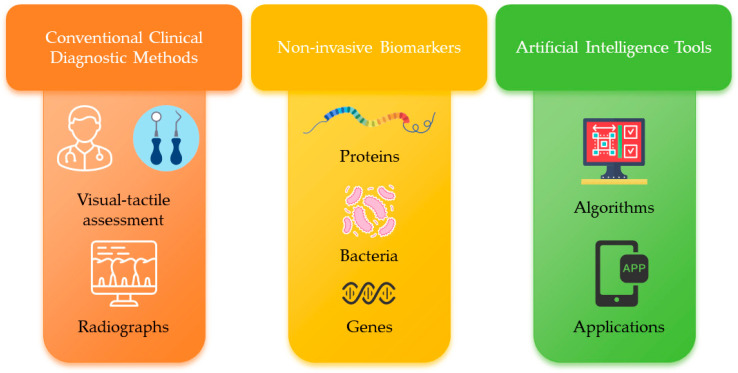
Diagnostic methods for detection of early childhood caries. ECC detection can be assessed using conventional clinical examination and radiographs, emerging non-invasive biomarkers, and AI-enabled decision-support tools.

Portable point-of-care devices and remote assessment platforms have also been proposed [47]. These tools aim to deliver rapid, chairside or home-based assessment. However, data on diagnostic accuracy, feasibility, cost-effectiveness, user acceptability, and integration into pediatric care pathways remain limited. Pragmatic, implementation-focused studies evaluating real-world performance are still scarce. Figure 3 is a schematic diagram of diagnostic methods for ECC detection.

## 4. Prevention and Implementation

### 4.1. Prevention Strategies

Effective strategies to manage ECC exist but face challenges in real-world adoption. Fluoride varnish applications [48], dental sealants [49], and caregiver education programs [50] are proven to reduce ECC. Despite established efficacy, consistent adoption in routine delivery settings remains limited.

A critical gap lies in scaling community-based efforts. School fluoride varnish programs, for instance, show promise but require consistent funding, trained staff, and parental consent [3]. In many regions, logistical hurdles like irregular school attendance derail implementation. Similarly, water fluoridation is a cost-effective public health measure, but it faces resistance due to misinformation about safety. Utah has become the first US state to ban the use of fluoride in its public water in 2025 [51]. Opposition groups often amplify unfounded claims, delaying or blocking policies that could benefit high-risk populations. Research rarely examines how to counter these narratives or build community trust in evidence-based ECC management.

### 4.2. Implementation Science

Implementation barriers are often amplified by health-system fragmentation. Even when policies like sugar taxes or fluoridation mandates pass, enforcement may be weak [37]. Studies are needed to identify which policies deliver the greatest impact for the lowest cost, particularly in resource-limited settings.

Cost-effectiveness and delivery-model comparisons remain limited, particularly under routine service conditions. Behavioral science approaches, such as nudging parents to improve children’s oral health, could improve adherence to dietary guidelines. Evidence remains limited regarding whether specific, theory-informed behavior-support components, such as environmental cues or digital reminders, produce sustained and scalable dietary change within routine implementation settings [52]. By addressing both systemic barriers and human behaviors, researchers can turn proven strategies into lasting solutions for ECC.

### 4.3. Behavioral and Psychological Interventions

Behavioral and psychological factors play a central role in ECC prevention, yet interventions targeting these areas remain underdeveloped [53]. Caregiver knowledge about oral hygiene, dietary habits, and access to dental care significantly shapes a child’s ECC risk [50]. Evidence is limited on which behavior-change components remain effective across contexts and over time. Traditional counseling in clinics often lacks follow-up, leading to short-term compliance that fades over time [54].

A critical gap is the limited evidence on digital tools designed to sustain behavior change. Mobile apps that track brushing frequency or send reminders about dental visits could reinforce healthy habits [55]. However, few studies test their effectiveness, particularly in low-literacy populations. Similarly, the role of parental mental health in ECC is overlooked. Stressed or depressed caregivers may struggle to maintain consistent oral care routines, yet dental programs rarely screen for or address these psychological barriers. Research is needed to understand how parental well-being directly influences a child’s oral health outcomes [56].

Gamification-based approaches have limited evidence beyond early feasibility studies, and generalizability across settings remains unclear [55,56]. Culturally adapted alternatives, such as sticker charts or community-based games, could offer low-cost solutions but require rigorous testing. Future interventions should prioritize community health worker programs to bridge gaps in access. Community health workers trained in oral health can deliver personalized counseling during home visits, addressing misconceptions about fluoride or sugar intake [32]. Evidence remains limited on scalable delivery supports that integrate behavioral, psychosocial, and service components without increasing inequities.

## 5. Treatment Modalities, Access and Equity

### 5.1. Treatment Modalities

Management of established ECC encompasses non-operative, minimally invasive, and restorative approaches. Non-operative strategies, including silver diamine fluoride (SDF), aim to arrest lesion progression without removal of carious tissue and may be particularly relevant for very young children and resource-constrained settings [57]. SDF has demonstrated effectiveness in arresting dentin caries in primary teeth; however, uncertainties remain regarding optimal application frequency, long-term durability of arrest, retreatment patterns, and comparative effectiveness relative to restorative options in routine clinical conditions [58]. Evidence linking arrest-based strategies to sustained disease control beyond short follow-up periods remains limited.

Minimally invasive and restorative treatments, such as atraumatic restorative treatment (ART), conventional restorations, stainless steel crowns, and the Hall technique, are widely used in primary dentition [59]. While controlled studies report favorable outcomes for selected techniques, comparative effectiveness data across diverse healthcare systems and risk profiles remain insufficient. Long-term restoration survival, retreatment burden, child-centered outcomes, and cost-effectiveness in preschool populations are inconsistently reported [60]. In advanced disease involving pulpal pathology, pulp therapy and treatment under general anesthesia are frequently required, yet recurrence rates remain high among high-risk groups [61,62].

A further gap concerns the integration and sequencing of treatment modalities. It remains unclear how arrest strategies such as SDF should be optimally combined with restorative interventions over time, particularly in children with persistent risk exposure. Real-world effectiveness studies and pragmatic trials are needed to clarify durability, recurrence patterns, and long-term disease trajectories across treatment pathways. Without such evidence, clinical decision-making remains guided primarily by short-term outcomes rather than sustained disease control.

### 5.2. Treatment Access and Equity

**Figure 4:**
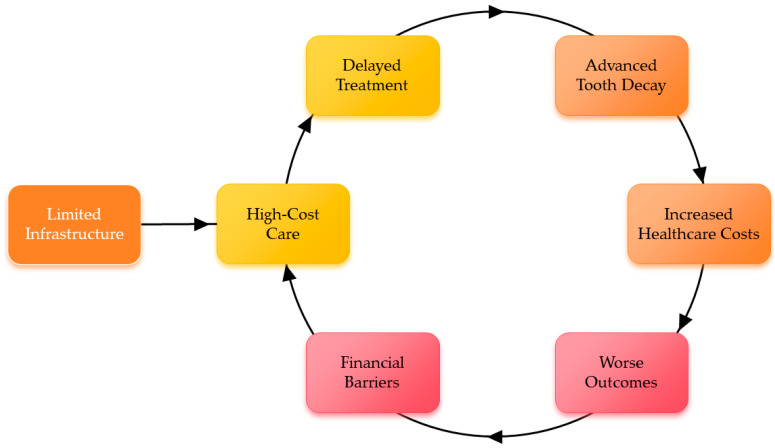
Cycle of early childhood caries treatment inequity. Limited infrastructure and financial barriers reinforce a self-perpetuating cycle of delayed, high-cost care that leads to advanced decay, worse outcomes, and rising healthcare costs.

Ensuring equitable access to effective treatments remains a major challenge in addressing ECC [32]. Restorative care under general anesthesia is a common solution for severe cases, but it requires specialized facilities and trained providers. In low-income regions, limited infrastructure and high costs force families to delay care, leading to worsened outcomes [7]. Even in wealthier areas, marginalized groups face long wait times and financial barriers. This imbalance perpetuates cycles of poor oral health, particularly among children from disadvantaged backgrounds. Figure 4 is a flow diagram showing the cycle of ECC treatment inequity.

Building on the above evidence, future strategies could focus on expanding access through delivery and financing reforms. Training mid-level providers, such as dental therapists or nurses, to apply SDF or sealants in schools or community centers may bridge gaps in rural areas [63]. Policy reforms are equally vital. Inclusion of essential oral health services for young children within universal health coverage benefits packages could reduce financial barriers, although the impact would depend on implementation design and available workforce capacity [32].

Several programs provide illustrative examples of delivery models that aim to improve equity. Thailand has successfully integrated basic dental services into national health systems, improving equity [2]. Smiling Brazil Program provides mobile dental units and teledentistry consultations could further reach underserved populations, though these require sustainable funding [64]. Cambodia’s Healthy Kids Cambodia delivered a school-based ECC package with tiered care guided by simple screening criteria, including supervised fluoride toothbrushing and SDF for caries arrest, to improve access for underserved children [65]. In developed settings, Scotland’s Childsmile program combines supervised toothbrushing in early years settings with targeted preventive support to reduce inequalities in child oral health [66]. Australia’s Smiles 4 Miles embeds oral-health-promoting policies and practices in childcare and kindergarten services, particularly in higher-risk communities [67]. Table 1 summarizes the barriers and strategies for improving children’s oral health.

Partnering with local leaders and schools to deliver these messages can build trust. Additionally, future research should explore innovative financing, such as microinsurance schemes or sliding-scale fees, to make care affordable. Addressing treatment inequity demands collaboration. More broadly, reducing treatment inequity will likely require coordinated action across government, insurers, and dental associations to address structural barriers to prevention and timely care. By prioritizing accessible, low-cost interventions and policy changes, the dental community can ensure all children—regardless of geography or income—receive timely care to prevent ECC’s lifelong consequences.

## 6. Long-Term Health and Developmental Outcomes

ECC has ripple effects on overall health and development. Studies link severe ECC to poor physical growth, likely due to chronic pain affecting nutrition and sleep [68,69]. Cognitive delays are also reported, possibly from prolonged inflammation or missed school days [70]. However, evidence generally comes from short-term studies, leaving long-term consequences poorly defined. Oral infections may trigger systemic inflammation, but this connection lacks robust longitudinal data. A critical gap is understanding how ECC influences lifelong health trajectories. Severe tooth decay in early childhood could damage developing adult teeth or jaw structures, increasing orthodontic needs [71]. Psychosocial impacts are understudied. Children with visible decay or tooth loss often experience bullying, leading to low self-esteem [72]. Anxiety around dental visits or speech difficulties from missing teeth may further hinder social and academic development. These effects could persist into adulthood, shaping career opportunities or mental health, yet research rarely follows ECC patients beyond adolescence [73].

Future research must prioritize long-term cohort studies tracking ECC survivors into adulthood. These studies should monitor systemic health markers, such as inflammatory cytokines or metabolic profiles, alongside dental outcomes. Including socioeconomic factors like income levels could reveal how ECC perpetuates cycles of disadvantage. Additionally, existing quality-of-life metrics focus on adults or general health, missing nuances specific to children [74]. Developing pediatric oral health tools that assess pain, social interactions, and school performance would provide a clearer picture of ECC’s true burden. Addressing these gaps requires collaboration between dentists, pediatricians, and public health experts. Integrating oral health assessments into routine pediatric checkups could help identify at-risk children early. Policies advocating for school-based dental screenings or nutritional programs might mitigate both immediate and downstream harms. By illuminating the lifelong shadow of ECC, researchers can push for interventions that foster the child’s oral and general health.

## 7. Global and Regional Disparities

ECC burden is unevenly distributed across countries and within populations, with many low- and middle-income countries (LMICs) reporting a higher prevalence of disease and a larger share of untreated lesions [8]. The World Health Organization (WHO) estimates over three quarters of untreated caries in primary teeth occur in LMICs. Between 1990 and 2019, global case numbers of ECC increased overall, with the sharpest increases in low-income and lower-middle-income countries despite a slight decline in average prevalence [75]. However, differences in ECC should not be explained by national income level alone. Pediatric dental coverage and delivery models vary substantially across settings. Some LMICs provide free or subsidized pediatric dental services through primary healthcare, while some high-income countries do not offer universal free dental care for children [76]. For this reason, disparities are better framed in terms of effective access, which may be constrained by geographic reach, service capacity and quality, affordability, and cultural acceptability, even where services nominally exist. Despite substantial burden in LMICs, most ECC research originates from wealthy nations, leaving critical gaps in understanding context-specific drivers in LMICs [77]. ECC and cultural norms are rarely studied. Furthermore, rural areas often lack basic dental infrastructure. Therefore, families need to travel long distances for care or rely on emergency treatments [78].

A key research gap lies in identifying how urbanization and globalization reshape ECC risks. Rapid dietary transitions in LMICs, such as increased availability of sugary beverages, are poorly documented [37]. Urban migrants may adopt Western-style diets high in refined sugars while retaining traditional oral hygiene practices ill-suited to these foods. This mismatch could accelerate decay, but few studies explore these interactions. Additionally, misinformation about fluoride or dental care often spreads faster than public health campaigns in regions with limited education resources, worsening disparities [79]. Another understudied area is the role of climate change and resource scarcity. Droughts or crop failures may push families toward cheaper, sugary alternatives to traditional diets. In conflict zones or refugee camps, disrupted water supplies and overcrowding heighten ECC risks by limiting access to clean water for oral hygiene [80]. Research rarely examines how such systemic shocks intersect with oral health outcomes, particularly for children.

Future efforts should prioritize cross-country comparisons to uncover effective, adaptable strategies. For instance, comparing regions with similar cultural practices but differing rates of ECC could reveal protective factors, like locally available anticariogenic foods. Partnerships between researchers, governments, and non-governmental organizations are also critical to integrate oral health into primary care systems in LMICs [81].

Training community health workers to apply fluoride varnish during routine child wellness visits could bypass dental workforce gaps. Cost-effective mobile clinics or school-based sealant programs tailored to regional needs could further reduce barriers [82]. Empowering LMIC researchers to lead studies ensures interventions align with local realities [80]. Global funding agencies should prioritize grants that support homegrown solutions rather than importing high-income country models. By addressing these disparities, the global health community can shift from a one-size-fits-all approach to strategies that respect cultural, economic, and environmental diversity. Table 2 summarizes the unaddressed questions and the proposed solutions for ECC management.

## 8. Interdisciplinary Collaboration

ECC is a complex public health issue influenced by biological, behavioral, and social factors [32]. Despite its multifaceted nature, research and clinical efforts often operate in isolation, limiting progress. Dentists typically focus on treatment, while pediatricians address general health, and public health experts design population-level interventions. This siloed approach overlooks opportunities to address shared risk factors, such as poor nutrition or limited healthcare access, that cut across disciplines.

Table 3 summarizes the research gaps of ECC. A major gap is the lack of integration between dental, medical, and social services. Many children visit pediatricians regularly but miss dental check-ups due to cost or caregiver unawareness. Pediatricians could screen for early caries during wellness visits, but most lack training in oral health assessment. Similarly, community programs targeting food insecurity or parental education seldom incorporate oral hygiene messaging.

Non-dental professionals, like social workers or lactation consultants, are untapped resources for promoting preventive practices. Without coordinated systems, families navigate fragmented care, delaying interventions until cavities become severe. Another challenge is the absence of unified guidelines for managing ECC across professions. Dental associations and pediatric groups often publish conflicting recommendations, confusing caregivers and providers [50].

Standardizing evidence-based protocols could align efforts, ensuring consistent messaging about brushing routines, diet, or fluoride supplements. Training programs must also evolve to foster collaboration. Medical students rarely receive oral health education, while dental curricula underemphasize social determinants of health [83]. Joint workshops for dentists, pediatricians, and community health workers could build mutual understanding and shared strategies.

Future initiatives should prioritize policy reforms that incentivize cross-sector partnerships. Embedding oral health into maternal-child health programs or school-based initiatives could normalize prevention. Technology, like shared electronic health records, could improve communication between dental and medical providers, flagging high-risk children earlier. Breaking down silos requires cultural shifts within institutions. Funding agencies should prioritize interdisciplinary research teams, while professional societies could create joint task forces. By uniting diverse expertise, stakeholders can design holistic solutions that address ECC’s root causes and not its symptoms.

## 9. Conclusions

In conclusion, ECC remains a preventable yet unequally distributed disease shaped by interacting biological, social, diagnostic, and system-level determinants. This review underscores that progress is constrained by persistent gaps in causal integration, limited validation of emerging detection and risk-assessment tools, and insufficient attention to equity in implementation. Addressing these gaps through integrated, context-sensitive research frameworks is essential for designing more coherent prevention, service delivery, and policy strategies that can genuinely reduce disease burden and disparities. These priorities are particularly relevant to researchers, clinicians, and policymakers seeking more effective, system-aware approaches to ECC prevention and management.

## Figures and Tables

**Table 1:** Barriers and Strategies for improving children’s oral health.

Barriers to Equitable Early Childhood Caries Treatment		
Category	Key Challenges	Impact [Reference no.]
Infrastructure Limitations	Limited access to general anaesthesia facilities.	Delayed care leading to advanced decay causing pain and spread of infection [7].
Financial Hurdles	High out-of-pocket costs and exclusion from insurance plans.	Care avoidance resulted in worsened oral health in marginalized groups [32].
Cultural Perceptions	Preference for aesthetics over silver diamine fluoride (SDF) therapy.	Underutilization of cost-effective care with SDF therapy [3].
**Strategies and success models for ECC management**		
Strategy	Key requirement	Success model [Reference no.]
Universal Health Coverage	Government advocacy to promote oral health.	Thailand’s integrated dental services: include basic care in national plans [2].
Mobile Dental Care Units	Sustainable funding models for dental care.	Brazil’s Smiling Brazil Program: offers teledentistry for remote diagnosis [64].
School-Based ECC Package	School partnership with trained teams and reliable supplies.	Cambodia’s Healthy Kids Cambodia: delivers three levels of care based on simple screening criteria [65].
National prevention model	Universal supervised brushing with targeted fluoride varnish application.	Scotland’s Childsmile Program: shifts the balance of care from treatment to prevention [66].

**Table 2:** Main themes and proposed solutions for early childhood caries management.

Main Themes and Unaddressed Questions for Early Childhood Caries	
Theme	Unaddressed questions
Urbanization	How do Westernized diets interact with traditional oral care practices to accelerate early childhood caries?
Dietary Transitions	Limited data on sugary beverage consumption patterns in low- and middle-income countries undergoing rapid urbanization.
Misinformation	Spread of dental myths outpaces public health education in low-literacy regions, especially in low- and middle-income countries.
Climate/Conflict	Impact of droughts/conflict on dietary shifts toward cariogenic foods and oral hygiene access.
**Strategies and proposed solutions for early childhood caries**	
Strategy	Implementation examples
Comparative Research	Analyze regions with similar cultures but divergent rates of early childhood to identify protective factors such as local anticariogenic foods.
Healthcare Integration	Train community health workers to apply professional fluoride varnish during child wellness visits for prevention of early childhood.
Infrastructure	Deploy mobile clinics/school sealant programs tailored to low- and middle-income countries resource constraints.
Capacity Building	Fund L low- and middle-income countries-led research initiatives; prioritize grants for culturally adapted interventions.

**Table 3:** Current research gaps of early childhood caries.

Research Gap	Description
Microbiome Dynamics	Limited understanding of how specific microbial communities and their interactions with diet/host factors drive ECC progression.
Social Determinants	Insufficient research on how socioeconomic status, parental education, and cultural practices influence ECC risk and access to prevention.
Diagnostic Innovations	Lack of affordable, non-invasive diagnostic tools for early detection of caries in young children, leading to delayed interventions.
Prevention Strategies	Existing preventive strategies are not widely adopted due to inadequate knowledge of real-world barriers to their implementation at scale.
Health Outcomes	The long-term effects of ECC on systemic health, academic performance, and psychosocial well-being are underexplored.
Global Disparities	Sparse data on ECC prevalence, risk factors, and outcomes in low-resource settings, obscuring context-specific challenges and solutions.
Behavioral Interventions	Need for deeper investigation into culturally sensitive interventions for caregiver feeding and child oral hygiene behaviors.
Genetic Susceptibility	Incomplete mapping of how genetic predispositions interact with environmental exposures to influence caries development.
Treatment Equity	Ongoing inequities in access to restorative and preventive care due to geographic, financial, and systemic barriers.
Interdisciplinary Collaboration	Lack of integration across research and clinical disciplines impedes comprehensive approaches to ECC’s multifactorial origins.

## Data Availability

No new data were created or analyzed in this study.

## Abbreviations

The following abbreviations are used in this manuscript:

AI	Artificial intelligence
ECC	Early childhood caries
LMICs	Low- and middle-income countries
SDF	Silver diamine fluoride
WHO	World Health Organization
